# High-Yield Recovery of Antioxidant Compounds from *Bambusa chungii* Culms Using Pressurized Hot Water Extraction

**DOI:** 10.3390/antiox11112231

**Published:** 2022-11-12

**Authors:** Xianshuang Cao, Yaoyao Zhang, Hang Xun, Jin Wang, Feng Tang

**Affiliations:** Key Laboratory of National Forestry and Grassland Administration Beijing for Bamboo & Rattan Science and Technology, International Centre for Bamboo and Rattan (ICBR), Beijing 100102, China

**Keywords:** phenolic compounds, bamboo culms, antioxidant capacity, pressurized hot water extraction, response surface methodology

## Abstract

A large amount of waste from *Bambusa chungii* culms is generated from the bamboo pulping industry, causing disposal problems. Nevertheless, bamboo culms are a suitable source of functional ingredients, such as antioxidant compounds. However, because of the high compactness and tightness in their material structure, obtaining phytochemicals from bamboo culms using conventional organic solvent extraction methods can be inefficient. In this research, we developed a pressurized hot water extraction (PHWE) method to recover 19 target phenolic compounds from *Bambusa chungii* culms. The extracted compounds were determined by ultra-high performance liquid chromatography–quadrupole time-of-flight–mass spectrometry (UPLC–QTOF–MS). The antioxidant potential of the extracts was evaluated by 2,2-diphenyl-1-picrylhydrazyl (DPPH), 2,2′-Azino-bis(3-ethylbenzothiazoline-6-sulfonic acid) (ABTS), and ferric reducing antioxidant power (FRAP) assays. We investigated the effects of temperature, extraction time, and the material-to-liquid ratio on PHWE, and these parameters were optimized with a Box–Behnken design experiment and response surface tool. The optimal extraction condition was found at 170 °C, with a 1:30 g/mL material-to-liquid ratio and a 14 min extraction time. Following these optimal parameters, the total yield of target phenolic compounds (TYPC) reached 3.85 mg/g of raw material, and the half-maximal inhibitory concentrations (IC_50_) for the DPPH and ABTS tests were 94.7 mg/L and 21.88 mg/L, respectively. The FRAP value was 1.23 μmol FSE/mg of dried extract. A strong correlation between TYPC and the antioxidant activity of the extract was confirmed. The TYPC and antioxidant capacity of the optimal PHWE extract of the *Bambusa chungii* culms were both considerably higher than those of extracts obtained from conventional solvent extractions. These results indicated that PHWE is an excellent green technique for recovering phenolic compounds from bamboo culms, and the PHWE extracts of *Bambusa chungii* culms may be a good source of natural antioxidants.

## 1. Introduction

Due to safety concerns about synthetic antioxidants in recent years, the use of natural antioxidants derived from plants has attracted considerable interest in the food, cosmetic, and pharmaceutical industries [1,2]. Despite decades of intense investigation, researchers are still seeking stable plant sources of natural antioxidants and highly efficient extraction technologies.

Bamboo, belonging to the Gramineae family, is widely distributed throughout Asia, Africa, and Latin America. Bamboo culms, as a fast-growing and renewable material, have been widely utilized for building material, furniture, flooring, and engineered composites [3]. The culms of some bamboo species are highly suitable for pulping and papermaking because of their anatomical structure, which includes the appropriate fiber length, fiber diameter, lumen diameter, and wall thickness [4,5]. In addition, bamboo plant-related herbal medicines have been used against oxidative stress-mediated diseases, such as cardiovascular and gastrointestinal diseases in China and India for thousands of years [6,7]. Phytochemistry studies validated that phenolic compounds are the potential active constituents for the pharmacological properties of bamboo [8,9,10]. In bamboo processing, especially the bamboo pulping industry, a certain amount of raw material from bamboo culms is discarded due to unsuitable fiber properties. Such waste is only partially valorized as animal feed or composting material at low value-added levels. Nevertheless, the culms could be inexpensive and abundant sources of high-value-added bamboo-derived functional ingredients, such as natural antioxidant compounds of vast interest to the pharmaceutical, cosmetic, and food industries. However, since bamboo culms are characterized by their compact, dense material structure and strong cell walls [11,12], obtaining ingredients from bamboo culms using conventional methods, such as organic solvent extraction under ambient conditions, requires a long operating period, exhibits poor extraction efficiency, and results in a low extraction yield. Therefore, there is a significant demand for effective extraction technologies to recover natural antioxidants from bamboo culms.

Pressurized hot water extraction (PHWE), also called subcritical water extraction or superheated water extraction, is an extraction technology that employs liquid water as the extraction solvent and requires elevated temperature and pressure conditions [13]. This method has attracted research interest for recovering bio-actives from plant materials in both laboratory and industrial processes. PHWE has many attractive features, including “green extraction”, a high recovery yield, a low operation cost, and, most importantly, extracted phytochemicals that can be used directly without the need for further cleanup or any safety concerns about the residual organic solvents [14,15,16].

Due to some unique advantages, PHWE could be considered a powerful approach for obtaining natural products from compact and dense plant tissues, such as bamboo culms. For instance, applying elevated temperatures and pressure improves the extraction efficiency, as these conditions disrupt the target substance–sample matrix interactions caused by van der Waals forces, hydrogen bonding, and dipole attraction [17]. Furthermore, the surface tension of the extraction systems decreases and therefore enhances the solvent wetting of the sample, allowing the target substance to dissolve in the solvent more sufficiently [18]. Finally, an important advantage of using higher temperature and pressure during extraction is the thermal energy and exerted pressure on the plant tissues, which disrupt the cellular structure and enhance the mass transfer of the target substance from the inner part of plant matrices to the solvent [19]. The extraction temperature is the most critical factor in the PHWE process. Since the temperature increases, the water dielectric constant decreases dramatically, affecting its polarity and dissociation constant [20]. Detailed information regarding the principles and mechanism of PHWE, especially the impact of elevated temperature, has been well described [21,22,23].

Numerous studies have demonstrated that phenolic compounds from various plant materials can be efficiently extracted using the PHWE method, and these procedures have been systematically investigated [24,25,26]. The common conclusion presented in these studies was that, compared to traditional extraction methods, PHWE approaches were preferred with higher recoveries, while being time and cost efficient. In some works, researchers found that when the extraction conditions were optimized by spectrophotometric methods, such as total phenolic and antioxidant assays, greater antioxidant capacities and higher total phenolic contents were observed in the extracts obtained at the highest investigated temperatures and longest extraction times [27,28]. These conditions would not be suitable for recovering all types of natural phenolic compounds since they are thermolabile compounds. The use of high temperatures and long extraction times could lead to the degradation of phenolic compounds and undesired reactions during extraction procedures [29,30]. New compounds formed in those reactions might result in erroneous spectrophotometric assay results for determining the recovery of total phenolic compounds. More specialized analytical techniques are necessary to qualify and quantify the active ingredients under optimal PHWE conditions.

*Bambusa chungii* is one of the most important sympodial bamboo species in China, and its culms are used as raw material for pulping and papermaking in vast quantities. It can also be applied to the preparation of traditional Chinese medicine [31]. We aimed to evaluate the technological potential of using PHWE to extract antioxidant compounds from *Bambusa chungii* culms. In this context, the extraction yield and total phenolic content (TPC) in the PHWE extracts of *Bambusa chungii* culms obtained at different temperatures were examined. We confirmed the occurrence of the Maillard reaction during the PHWE process. The extracted compounds were identified and quantitatively determined through ultra-high performance liquid chromatography–quadrupole time-of-flight–mass spectrometry (UPLC–QTOF–MS). We systematically determined the effects of temperature, extraction time, and material-to-liquid ratio on the recovery of phenolic compounds and the antioxidant capacity of the extracts. The PHWE process was optimized using response surface methodology (RSM) to obtain a maximum total yield of targeted phenolic compounds with simultaneously good antioxidant properties of the extracts. We compared the PHWE method with the conventional extraction methods in terms of the extracts’ antioxidant properties.

## 2. Materials and Methods

### 2.1. Chemicals and Reagents

HPLC grade methanol, ethanol, and acetonitrile were purchased from Thermo Fisher Scientific (Waltham, MA, USA). Standards of phenolic compounds, including, quinic acid, caffeic acid, vanillic acid, syringic acid, ferulic acid, arbutin, adenosine, tachioside, vanillic alcohol, *p*-hydroxybenzoic acid, *p*-hydroxybenzaldehyde, vanillin, *p*-coumaric acid, coniferol, syringaldehyde, acetovanillone, sinapic acid, coniferylaldehyde, sinapaldehyde, syringaresinol, and balanophonin; standards of furfural (FF) and 5-hydroxymethylfurfural (HMF) were obtained from Sigma-Aldrich (MO, USA) with purity ≥ 98%. Folin–Ciocalteu (FC) reagent, 2,2-di(4-tert-octylphenyl)-1-picrylhydrazyl (DPPH), 2,2′-azino-bis(3-ethylbenzothiazoline-6-sulfonic acid) (ABTS) were provided by Yuanye (Shanghai, China). FRAP test kit was purchased from Solibao (Beijing, China). Other chemicals (analytical grade) were supplied by Sinopharm (Beijing, China). Purified water was obtained using a PALL Lab Water Purification System (Port Washington, NY, USA).

### 2.2. Preparation of Materials

*Bambusa chungii* culms were provided by Jinhua Bamboo Paper-making Company, the bamboo plants were collected from Yang Jiang City, Guangdong Province, China. The bamboo culms were cut into small chips ranging in size from 1.0–4.0 cm, and without further being powdered. This material preparation was according to the form of waste generated from the bamboo pulping mill. The moisture content of the bamboo chips was 31.25%. The bamboo chips were stored at −20 °C until they were used.

### 2.3. Pressurized Hot Water Extraction

The pressurized hot water extraction was carried out in a batch-type extractor (HT-1000FJ, Huotongyiqi, Shanghai, China), the apparatus mainly consisted of a 1 L agitated controlled autoclave installed in a heating mantle (Figure S1). In a typical experiment, the *Bambusa chungii* culm chips and pure water were loaded into the extractor, after being tightly closed with stainless steel cap, the extractor was placed in the heating mantle, and heated up to the desired temperature. The internal temperature of the extractor was measured using a thermocouple device. During the process, a 500 rpm agitator continuously agitated the extract. After extraction, the extractor was removed from the heating device and cooled at room temperature. The extract was filtered through qualitative filter paper (No. 1 Whatman, Maidstone, UK), and the filtrate was stored at −20 °C for further experiments.

The effects of temperature (100, 120, 140, 160, and 180 °C), material-to-liquid ratio (1:5, 1:10, 1:15, 1:20, and 1:30 g/mL), and extraction time (5, 10, 15, 20, and 30 min) on PHWE extraction were investigated with single-factor experiments. Each parameter was optimized in triplicate experiments, while the other two parameters were kept constant as follows: the temperature was set to 160 °C, the material-to-liquid ratio was set to 1:20 g/mL, and extraction time was set to 20 min.

A certain volume of filtrate from each extracted sample was evaporated, then dried further in a freeze dryer for 1 day after being frozen in a −20 °C freezer. The powder products were weighed, and those data were used for extraction yield calculation (Equation (1)).

Extraction yield (%) = m_Extract_/m_RM_ × 100 (1)

where m_Extract_ is the mass of the dried extract and m_RM_ is the mass of the initial raw material.

To estimate the formation degree of browning compounds, such as melanoidins in the extracts, the optical density of filtrate from each extracted sample was measured at 420 nm (OD_420_) using a spectrophotometer reader (UV-2100, PerkinElmer, Waltham, MA, USA) [32].

### 2.4. Conventional Extractions

Maceration and reflux extraction methods were implemented to obtain comparative data for PHWE extraction. Two conventional extraction procedures were performed as described by Nuzul et al. and QIN et al. for *Bambusa beecheyana* culms [33] and *Bambusa surrecta* culms [34], respectively, with modifications. Maceration was conducted by placing 30 g of bamboo culm chips into a distillation flask followed by the addition of 80% (*v*/*v*) ethanol 600 mL. The mixture was stood for 72 h at room temperature. This procedure was repeated twice. The reflux extraction method was carried out using 80% (*v*/*v*) ethanol at 80 °C for 2 h and repeated for three times. In each conventional extraction, the liquid extracts were collected and combined, then filtered through a filter paper under vacuum. Filtrates were concentrated to a constant volume (600 mL) using a rotary vacuum evaporator (Rotavapor R-215, Buchi, Flawil, Switzerland) at 50 °C. The concentrated filtrates were collected and stored at −20 °C for use in further analysis. The measurements of extraction yield and OD_420_ were also conducted according to the methods interpreted above.

### 2.5. Quantification of Total Phenolics

Total phenolic content was determined by the Folin-Ciocalteu assay [35,36], with minor modifications. 0.5 mL diluted extract was mixed with 2.0 mL Folin-Ciocalteu reagent (1 M) and incubated for 3 min. Then, 1.5 mL of 1% (*w*/*v*) saturated sodium carbonate solution was added to the mixture and placed in the dark for 2 h at room temperature. The absorbance of this mixture was read at 765 nm using the spectrophotometer mentioned above. The amount of total phenolics was calculated as milligram of gallic acid equivalents (mg GAE) per g of *Bambusa chungii* culms (raw material) based on a standard curve for gallic acid solution with concentrations from 1.5–12.5 μg/mL.

### 2.6. Identification and Quantification of Individual Phenolics

The phenolic compounds, FF and HMF were identified and quantified, as described by the authors elsewhere [37,38], by employing an Agilent 6540 quadrupole time-of-flight mass spectrometer using electron spray ionization (ESI-QTOF-MS) coupled to an Agilent 1290 ultra-high performance liquid chromatography (UPLC) system equipped with a diode array detector (DAD) (Agilent Technologies, Santa Clara, CA, USA). The dried extracts were resuspended in 30% (*v*/*v*) methanol solution and filtered through a 0.2 μm hydrophilic membrane (Merck, Burlington, MA, USA) before injection into the UPLC-QTOF-MS.

The LC separation was conducted on a ZORBAX Eclipse Plus C18 column (1.8 μm, 2.1 mm × 150 mm, Agilent Technologies, Santa Clara, CA, USA) at 40 °C. The mobile phase was a six-step linear gradient prepared from 0.1% formic acid aqueous solution (mobile phase A) and acetonitrile (mobile phase B). The starting composition was 95:5 (% *v*/*v*) mobile phase A-B, and acetonitrile content was increased to 90% over a 55 min period. The composition of the gradient is reported in Table S1. The mobile phase flow rate was 0.25 mL/min. UV visible spectra were recorded in the range 190–450 nm and chromatograms were acquired at 210, 254, 280, and 320 nm.

Identification of individual compounds was achieved by use of their retention times and mass spectrum data. The TOF-MS system was operated in positive mode and the mass analysis conditions were set as follows: drying gas (N_2_) flow rate, 7 L/min; drying gas temperature, 350 °C; fragmentor voltage, 40 V; nebulizer, 35 psi; capillary voltage, 4500 V; electrospray spray voltage, 3500 V; sheath gas temperature, 350 °C; nozzle voltage, 1000 V; Mass spectra were acquired in the m/z range of 50 to 800. The MS data were collected in a MS scan mode. System control and data analysis were performed using the software MassHunter 8.0.0 (Agilent Technologies, Santa Clara, CA, USA).

### 2.7. Evaluation of Antioxidant Capacity

#### 2.7.1. Ferric Reducing Antioxidant Power (FRAP) Assay

The FRAP assay was performed according to the instruction of the FRAP test kit (Solibo, Beijing, China). This method is based on the reduction of colorless ferric complex (Fe^3+^-TPTZ) to the blue-colored ferrous form (Fe^2+^-TPTZ) by the action of electron-donating antioxidants at low pH [39]. The FRAP reagent was prepared with a 300 mmol/L acetate buffer (pH 3.6), 20 mmol/L ferric chloride, and 10 mmol/L TPTZ (in 40 mmol/L hydrochloric acid) at a ratio of 10:1:1. 24 μL of the extract was mixed with 180 μL of FRAP reagent and the reaction mixture was incubated at 37 °C for 10 min. The absorbance was read at 593 nm using a multilabel plate reader (Victor 2030, PerkinElmer, Waltham, MA, USA). A calibration curve was plotted with ferrous sulphate (FeSO_4_·7H_2_O) used as standard (linearity range: 0.00625–0.15 μmol/mL, *R*^2^ = 0.9999). Results were expressed in µmol ferrous sulphate equivalents (FSE) per milligram of extract on dry weight (DW).

#### 2.7.2. DPPH Radical Scavenging Assay

The DPPH assay was conducted as Scherer et al. described [40], with minor modifications. Extract samples were diluted to final concentrations in ethanol. Each diluted sample of 1 mL was mixed with 2 mL of 50 mg/L DPPH ethanolic solution at room temperature. After 30 min of reaction protected from light, the absorbance was measured at 517 nm using a UV–vis spectrophotometer mentioned above. The tests were accompanied by a control (ethanol with DPPH reagent) and the sample blanks (sample with ethanol, without the DPPH reagent). The scavenging percentage of DPPH radical was calculated as followed:

Scavenging percentage of DPPH (%) = [1 − (A _sample_ − A _blank)_/A _control_] × 100% (2)

where A_sample_: test sample with DPPH reagent absorbance; A_blank_: blank absorbance; and A_control_: control absorbance. The concentration of extracts required to quench 50% of the DPPH radical under the assay conditions was determined from dose–response curves. The result of the testing sample was expressed as half maximal inhibitory concentration (IC_50_) value (μg/mL).

#### 2.7.3. ABTS Radical Scavenging Assay

ABTS assay was done using the modified methods from Re et al. and Kenny et al. [41,42]. ABTS solution (7 mM) was mixed with potassium persulfate (2.45 mM) and incubated in the dark for 16 h to form ABTS radical cations (ABTS^•+^). This solution was diluted with ethanol until reaching an absorbance of 0.70 ± 0.05 at 734 nm. A total of 2.0 mL of this solution was mixed with 0.5 mL of test sample. The mixture was vortexed and incubated for 6 min in a dark condition. Test sample mixed with ethanol was used as the blank, and diluted ABTS^•+^ solution without mixing the test sample was used as the control. The absorbance was measured with the same UV–vis spectrophotometer described above at 734 nm. The ABTS scavenging percentage of each sample was estimated according to Equation (3). IC_50_ values were calculated using the dose–response model.

Scavenging percentage of ABTS (%) = [1 − (A_sample_ − A_blank_)/A_control_] × 100% (3)

where A_sample_: test sample with ABTS^•+^ solution absorbance; A_blank_: blank absorbance; and A_control_: control absorbance. The result of the testing sample was expressed as ABTS IC_50_ value (μg/mL).

### 2.8. Design of Experiments

A 3-factor 3-level Box–Behnken factorial experimental design was applied in the RSM model to observe the individual and combined effect of temperature, extraction time, and material-to-liquid ratio on the total yield of target phenolic compounds and antioxidant activities (measured by DPPH, ABTS, and FRAP assays) of the extracts, as well as to find the optimum extraction conditions. Three levels of each variable were tested, and the values were set according to the results of the preliminary single-parameter screening. A total of 17 experimental runs were performed in a randomized manner. The Design-Expert software (7.1.6 Stat-Ease Inc., Minneapolis, MN, USA) was used to design and evaluate the three independent variables at three levels on the responses according to Equation (4):

(4) $$Y_{i}=\beta_{0}+\underset{i}{\sum }\beta_{i}{Χ}_{i}+\underset{ii}{\sum }\beta_{ii}{Χ}_{i}^{2}+\underset{ij}{\sum }\beta_{ij}{Χ}_{ij}$$

where *Y_i_* is the investigated response variable, *X_ì_* and *X_j_* are investigated independent variables, *β_0_* is the constant coefficient (intercept), while *β_i_*, *β_ii_*, and *β_ij_* are the linear, quadratic, and interaction coefficient, respectively.

### 2.9. Statistical Analysis

All experiments were conducted in triplicate, and the results were expressed as mean values ± standard deviation (SD). Data were statistically analyzed using SPSS software (20.0 SPSS Inc, Chicago, IL, USA). One-way Analysis of Variance (ANOVA) was employed to assess the statistical significance of the differences among multiple groups. Duncan’s multiple range tests were performed to determine significant differences between the mean values of the treatments (*p* < 0.05). Pearson’s correlation test was used for correlation analysis.

## 3. Results

### 3.1. Effects of Temperature on the Extraction Yield and Total Phenolic Content

As mentioned in the Introduction, our objective for this research was to develop an efficient pressurized hot water extraction method to recover phenolic antioxidants from bamboo culms. We were convinced that temperature was the most important parameter influencing the physicochemical properties of the whole PHWE system. For this purpose, the extraction yield and total phenolic contents were chosen as indicators to investigate the extraction abilities of PHWE at different temperatures.

Table 1 shows data for the extraction yields and total phenolic contents using PHWE at 100 to 180 °C with a constant material-to-liquid ratio of 1:20 g/mL for 20 min. As the temperature increased within the range applied (100–180 °C), the extraction yield and total phenolic content also increased. The highest extraction yield (22.61%) and total phenolic content (7.6 mg GAE/g of raw materials RM) were obtained at 180 °C. Similar facts about the extraction yield and total phenolic content increasing with temperature have been extensively observed in PHWE studies and are explained by the increasing mass transfer, lower surface tension, and higher solubility of numerous compounds resulting from the increased temperature [43,44,45].

The application of PHWE at temperatures higher than 140 °C resulted in higher extraction yields and total phenolic contents than conventional extraction methods. The highest TPC value obtained in this work (7.6 mg GAE/g of plant material at 180 °C) was lower than the values for five native Brazilian bamboo species reported by Wroblewska et al. (43.64~87.81 mg GAE/g RM) [46] but higher than the values from bamboo culms and related products reported by Gomez et al. (1.59~3.84 mg GAE/g RM) [47], Gong et al. (4.21 mg GAE/g RM) [48], and Nuzul et al. (0.45~0.72 mg GAE/g RM) [33]. The literature data listed above (converted to the same unit) were all obtained from conventional extraction methods using organic solvents and measured by the FC method. Differences between the reported TPC values and our results may be attributed to the different raw materials. In any case, these comparisons suggest, in terms of the extraction yield and total phenolic contents, that PHWE is a superior alternative for obtaining phytochemicals from bamboo culms.

However, the high extraction yield and TPC obtained under high-temperature conditions may indicate that some undesired compounds were neo-formed via Maillard or other reactions and co-extracted. The development of a brown color in the extracts can be used as the simplest method to monitor the occurrence of Maillard and caramelization reactions during PHWE (the color of the extracts is presented in Figure S2) [27]. The browning intensity of the extracts measured at 420 nm is shown in Table 1, suggesting that the browning compounds were formed at approximately 140 °C and continuously increased with higher temperature. Another indicator commonly used to characterize the extent of the Maillard reaction is the formation of HMF and furfural [49]. HMF and furfural were not detected in the two extracts using conventional methods but were first detected in PHWE extracts at temperatures of 140 °C (HMF: 69 µg/g RM) and 160 °C (FF: 221 µg/g RM), respectively. Both compounds increased substantially with higher PHWE temperatures (HMF: 2246 µg/g RM and FF: 2805 µg/g RM at 180 °C). Maillard and caramelization reactions appeared alongside PHWE at high temperatures as deduced from the formation of a brown color, HMF, and furfural. This finding will likely affect the results of TPC measurements.

The Folin–Ciocalteu colorimetric assay is one of the oldest methods to determine the total phenol content. The methodology is based on transferring electrons from phenolic compounds and other reducing species to molybdenum; consequently, blue complexes are formed and can be detected spectrophotometrically [50]. The FC reagent is not specific to phenolic compounds. For that reason, the observed TPC increase in the extracts using a temperature higher than 140 °C in this research was probably due to contributions from new compounds formed in the Maillard and caramelization reactions, which are known for their potent reducing activity [49,51]. The qualitative and quantitative characteristics of the phenolic compounds obtained from the bamboo culms’ PHWE extracts should be analyzed using more accurate technology.

### 3.2. Effects of Single Extraction Parameters on the Recovery of Target Phenolic Compounds

**Figure 1:**
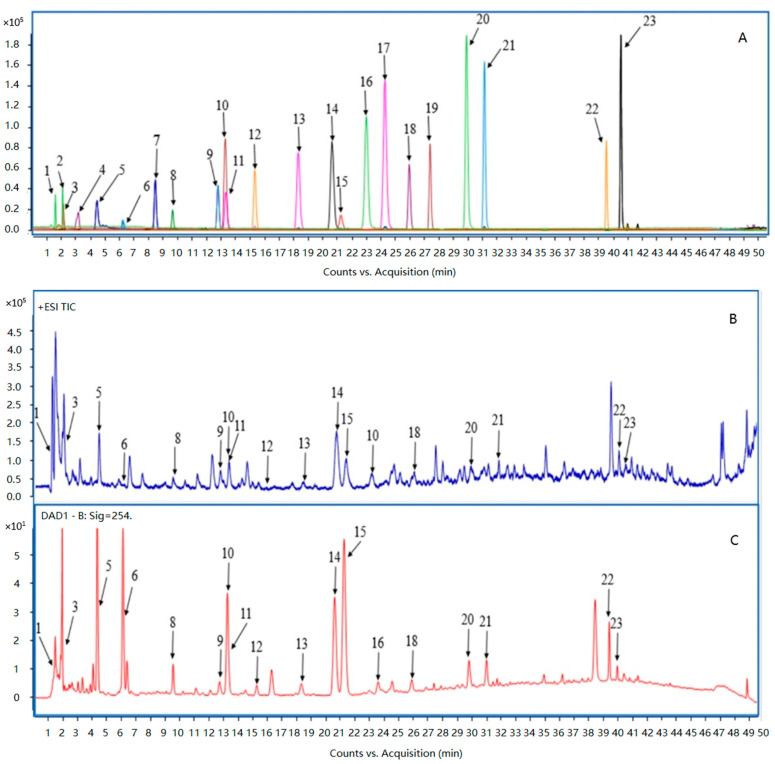
Liquid chromatography/quadrupole-time-of-flight mass spectrometry (LC-QTOF-MS) total ion chromatograms (TIC) of target compounds standards mixture (**A**), the PHWE extract of *Bambusa chungii* bamboo culm (**B**), and UPLC-DAD detection diagram of the PHWE extract of *Bambusa chungii* bamboo culm monitored at 254 nm (**C**). Peak identification:1. Quinic acid, 2. Arbutin, 3. Adenosine, 4. Tachioside, 5. 5-Hydroxymethylfurfural, 6. Furfural, 7. Vanillic alcohol, 8. *p*-Hydroxybenzoic acid, 9. Vanillic acid, 10. *p*-Hydroxybenzaldehyde, 11. Caffeic acid, 12. Syringic acid, 13. Vanillin, 14. *p*-Coumaric acid, 15. Coniferol, 16. Syringaldehyde, 17. Acetovanillone, 18. Ferulic acid, 19. Sinapic acid, 20. Coniferylaldehyde, 21. Sinapaldehyde, 22. Syringaresinol, 23. Balanophonin.

High-performance liquid chromatography (HPLC) is one of the most powerful methods for separating and monitoring mixtures of small molecules. Coupled with mass spectroscopy (MS), LC–MS is ideally suited for detecting and quantifying multi-components in complex plant extracts [52]. Based on our previous work on the chemical constituents of bamboo plants [38,53,54,55], 21 phenolic compounds were selected as the extraction targets in this research. The molecular structures of the target compounds are shown in Figure S3. The target compounds were identified by UPLC–QTOF–MS through comparing their retention times and accurate mass spectrometry data to reference standards. Detailed MS data are shown in Table S2. The chromatographic profiles of mixed reference standard compounds and the PHWE extracts of the *Bambusa chungii* culms are shown in Figure 1. According to the chromatograms, the target phenolic compounds were the primary constituents in the extracts.

The target phenolic compounds in the PHWE extracts obtained at different temperatures are listed in the elution order in Table 2. The contents of the target phenolic compounds obtained under the different extraction times and the material-to-liquid ratios are tabulated in Tables S3 and S4, respectively. According to the results, 19 target phenolic compounds were identified and quantified. Compared to the two extracts obtained from conventional methods, the PHWE extracts indicated evidently better results in obtaining target phenolic compounds from *Bambusa chungii* culms in terms of both variety and recovery yield. A few of the target phenolic compounds were detected only in the PHWE extracts, indicating that some phenolics originally linked to the structural components of the plant (cellulose, proteins, and lignin) or larger polyphenols might have been extracted by PHWE at elevated temperatures [56].

Regarding the phenolic compounds quantified in the PHWE extracts, the top three with the highest concentrations were coumaric acid, coniferol, and syringaresinol. The recoveries of these three compounds showed similar trends with changes in the extraction temperatures. When the temperature increased from 100 to 120 °C, a mild increase in recovery was observed. Then, the contents increased markedly in the temperature range of 120–140 °C, and reached their maximum concentrations (coumaric acid: 1145 µg/g, coniferol: 942 µg/g, and syringaresinol: 519.4 µg/g) at 160 °C. Further temperature increases resulted in the degradation of these three compounds. At 180 °C, the coumaric acid content decreased slightly to 1081 µg/g, the coniferol content dropped sharply by 94% to 49.5 µg/g, and the syringaresinol content decreased by 61% to 210 µg/g. The maximum recoveries of arbutin (14.4 µg/g), adenosine (31.9 µg/g), caffeic acid (13.5 µg/g), and vanillic alcohol (23.1 µg/g) were achieved at the moderately elevated temperatures of 120 or 140 °C before decreasing in the investigated temperature range. Due to the existence of glycosidic bonds, arbutin and adenosine are highly sensitive to the solubility, polarity of extraction solvents, and the extraction temperature [27,57]; therefore, they only appeared in the PHWE extracts obtained at a narrow temperature range of 100–120 °C. Under subcritical water conditions, caffeic acid quickly degrades into hydroxytyrosol, protocatechuic aldehyde, and 4-vinylcatechol; furthermore, DPPH assay confirmed that the degradation products retained good antioxidant activity [58]. This finding revealed an interesting fact about PHWE technology—even if the original compounds are degraded, extracts with high antioxidant capacity may be attainable by PHWE. The other 12 target phenolic compounds were stable in the operating temperature range explored in this research (100–180 °C), and their contents increased with the extraction temperature. Our finding suggested that, using the PHWE method, the recovery of different target phenolic compounds from *Bambusa chungii* culms was favored at different temperatures. Increasing the temperature above a certain value would result in decreased amounts of thermolabile compounds. The degradation of phenolic compounds in PHWE was strongly related to the compounds’ chemical structure. A systematic quantitative study on the decomposition kinetics of nine phenolic compounds under subcritical water conditions indicated that substituent groups promoted the thermal degradation of phenolic acids on the ring structure, such as amino, hydroxyl, and methoxyl [59]. Moreover, in a complex plant material PHWE system, the extracted compounds may participate in the reactions with other co-extracted ingredients and lead to decreased contents [60]. Further studies are needed to clarify the detailed extraction behavior and molecular transformation mechanism of individual target phenolic compounds in the PHWE process presented in this research.

**Figure 2:**
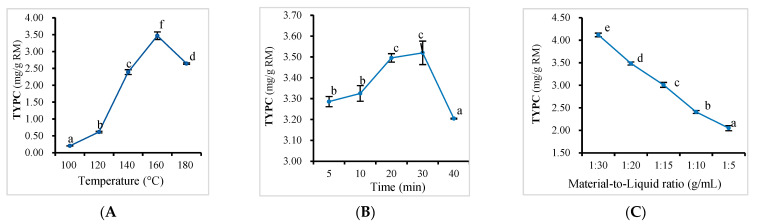
Effect plots of the temperature (**A**), extraction time (**B**), and material-to-liquid ratio (**C**) on the total yield of all identified 19 target phenolic compounds (TYPC) (mean ± SD; *n* = 3). Values represented with different lowercase letters are statistically significant differences (*p* < 0.05).

The total yield of all identified 19 target phenolic compounds (TYPC) was considered to evaluate the effect of single parameters, including the temperature, extraction time, and material-to-liquid ratio, on the PHWE efficiency of *Bambusa chungii* culms. The TYPC notably increased from 202.8 to 3470 µg/g with the increase in temperature from 100 to 160 °C and then decreased to 2644 µg/g at 180 °C (Figure 2A), mainly due to the degradation of coumaric acid, coniferol, and syringaresinol, the three major extracted compounds. The extraction time was another crucial parameter that directly affected the extraction efficiency and degradation degree of the extracted phytochemicals [19]. A longer extraction time would increase the efficiency of PHWE because of the sufficient solubilization of bioactive compounds. However, when the mass transfer reaches equilibrium, a prolonged extraction time does not improve the extraction yield and may cause the degradation of bioactive compounds induced by their thermal instability. As seen in Figure 2B and Table S3, when the extraction time extended from 5 to 30 min, the TYPC increased from 3287 to 3520 µg/g. However, there was no significant difference (*p* > 0.05) in the yield obtained at 20 (3496 µg/g) and 30 min (3520 µg/g). Subsequently, the yield began to decrease with an increase in the extraction time (3204 µg/g, at 40 min) because of the decomposition of already extracted compounds. The material-to-liquid ratio affected the solubility and partition–equilibrium constant of the PHWE process [22]. Figure 2C (and Table S4) shows that an increase in the material-to-liquid ratio from 1:30 to 1:5 g/mL led to a persistent decrease (4119–2050 µg/g) in the TYPC. However, when the material-to-liquid ratio was low, more impurities (non-target compounds) may dissolve, and subsequent purification would be required, leading to the degradation and loss of target compounds in the extract.

In summary, the PHWE method showed promising potential for recovering our target phenolic compounds from *Bambusa chungii* culms. The recovery was influenced by the temperature, extraction time, and material-to-liquid ratio parameters. Further optimization of the PHWE conditions is needed. The antioxidant activities of phenolic compounds are widely known [56,61,62], implying a solid molecular basis for the considerable antioxidant capacity of PHWE extracts from *Bambusa chungii* culms.

### 3.3. Effects of Single Extraction Parameters on the Antioxidant Capacity of PHWE Extracts

PHWE technology has been widely used to obtain relatively polar compounds (especially phenolic compounds) from various plant matrices. In our research, the PHWE extracts of the *Bambusa chungii* culms were rich in phenolic compounds. In past decades, phenolic compounds were thoroughly studied for their potential bioactive benefits as antioxidants. The structural composition of the phenolic compounds influenced their antioxidant activity. The hydroxyl substituents in aromatic rings produced stable semiquinone free radicals through the reaction of a phenolic hydroxyl group with free radicals, thus terminating the chain reaction of the free radicals. The free radical-scavenging activity of phenolic compounds was based on the number and position of hydroxy and methoxy groups. Combined with di-active groups, especially the hydroxyl group, the ortho position in an aromatic ring had high free radical scavenging activity due to the lower bond dissociation energies of the hydroxyl groups. In addition, the electron-donating ability of the carboxylic acid group in the phenolic acids also contributed to the antioxidant activity [63,64].

As it was difficult to measure the contribution of the antioxidant activity from individual target phenolic compounds, we evaluated the overall antioxidant capacity of *Bambusa chungii* culm extracts by the commonly-used methods of DPPH, ABTS, and FRAP assays. The effects of the temperature, extraction time, and material-to-liquid ratio were evaluated, and the correlation between the antioxidant capacity and the total yield of target phenolic compounds in the extracts was examined.

**Figure 3:**
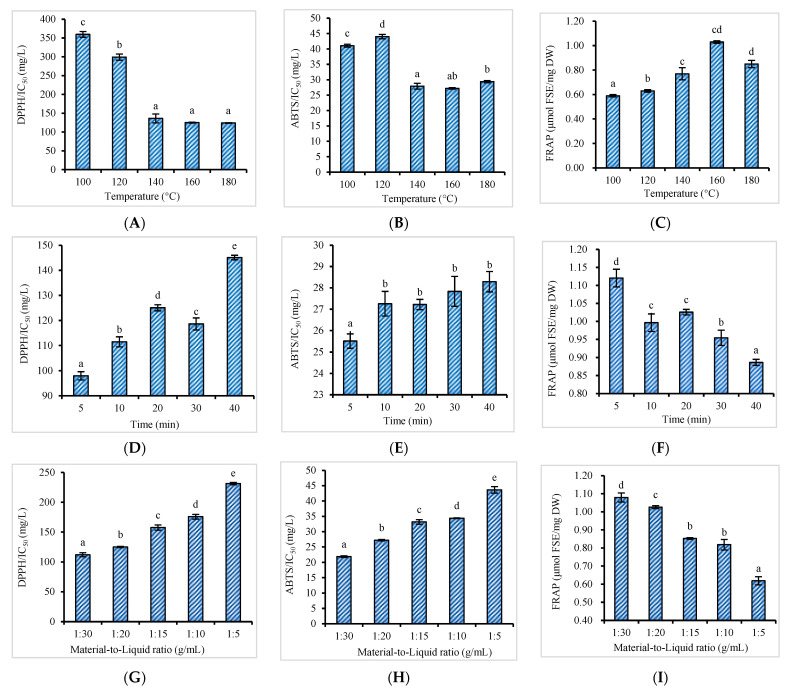
Effect plots of the temperature (**A**–**C**), extraction time (**D**–**F**), and material-to-liquid ratio (**H**,**I**) on the antioxidant capacity measured by 2,2-diphenyl-1-picrylhydrazyl (DPPH), 2,2′-azino-bis (3-ethylbenzothiazoline-6-sulfonic acid) (ABTS), and ferric reducing antioxidant power (FRAP) assays (mean ± SD; *n* = 3). Values represented with different lowercase letters are statistically significant differences (*p* < 0.05). DW: dry weight.

The antioxidant capacity of the PHWE extracts of the *Bambusa chungii* culms was affected by temperature, as seen in Figure 3A–C. Antioxidant activities increased with increasing temperature, and the values obtained over the range of 140–180 °C were notably higher than those from comparatively low-temperature (100 and 120 °C) extracts. The strongest antioxidant activities, as measured by DPPH (IC_50_: 125.07 mg/mL), ABTS (IC_50_: 27.22 mg/mL), and FRAP (1.03 μmol FSE/mg DW), were achieved at 160 °C. This result correlates well with the PHWE extracts of *Bambusa chungii* culms obtained at 160 °C, which had the highest amount of total target phenolic compounds. Interestingly, as the extraction temperature increased to 180 °C, the recovery of the total target phenolic compounds decreased due to the degradation of thermally unstable substances, which theoretically reduced the antioxidant capacity of the extracts. However, no significant differences in the antioxidant activities were observed between the extracts obtained at 160 and 180 °C. One possible explanation for this result was discussed previously: the enhanced formation of Maillard reaction products (MRPs) with potent antioxidant activity at high temperatures contributed to the antioxidant capacity of the extracts.

We performed Pearson’s correlation analysis between the antioxidant capacity and the total yield of the target phenolic compounds of the extracts prepared at different temperatures. The coefficients are summarized in Table 3. As we hypothesized, a highly significant linear correlation (*r*^2^ > 0.9) was found. This result indicated that the selected target phenolic compounds could be used to predict the antioxidant capacity of the PHWE extracts of *Bambusa chungii* culms.

Regarding the effect of the extraction time, our results (Figure 3D–F) indicated that an extraction process > 20 min did not increase the antioxidant capacity of the PHWE extracts. The extract obtained at the longest extraction time (40 min) had the lowest total yield of target phenolic compounds and the weakest antioxidant activity (DPPH IC_50_: 145.12 mg/mL, ABTS IC_50_: 28.29 mg/mL, and FRAP value: 0.88 μmol FSE/mg DW). It is worth noting that the extracts obtained at 5 and 10 min showed good antioxidant capacity: the DPPH IC_50_ values were 97.96 and 111.47 mg/mL; the ABTS IC_50_ values were 25.50 and 27.25 mg/mL; and the FRAP values were 1.12 and 0.99 μmol FSE/mg DW, respectively. This result is consistent with the literature: a higher antioxidant capacity was always observed in the PHWE extracts obtained at a comparatively short extraction time [65,66]. However, in this study, the extracts at 5 and 10 min did not show superiority in the amount of total target phenolic compounds. One possible reason could be that some unstable compounds, such as flavonoids and their glucosides, were co-extracted under the short extraction time and contributed to the antioxidant activity of the extracts [67].

Figure 3G–I shows the effect of the material-to-liquid ratio. The highest antioxidant activities of the PHWE extracts were achieved at the lowest material-to-liquid ratio. The DPPH IC_50_ was 112.08 mg/mL, ABTS IC_50_ was 21.89 mg/mL, and the FRAP value was 1.07 μmol FSE/mg DW. The antioxidant activity decreased as the material-to-liquid ratio increased, demonstrating a good linear relationship with the total target phenolic compound content. As discussed previously, although a lower material-to-liquid ratio favors the recovery of target compounds and the antioxidant activity of the extracts, a low material-to-liquid ratio could also have disadvantages in practical operability, which should be considered when optimizing the extraction process.

In summary, the temperature is the most influential parameter in the PHWE process. In the present research, the PHWE extracts of the *Bambusa chungii* culms obtained at temperatures higher than 140 °C exhibited high antioxidant capacities, and 160 °C was an inflection point for the recovery of the target phenolic compounds. Therefore, an extraction temperature that ranged from 140 to 180 °C, a comparatively short extraction time ranging from 5 to 20 min, and a moderate material-to-liquid ratio ranging from 1:15 to 1:30 were selected as the values for extraction parameter optimization.

### 3.4. Optimal PHWE Conditions

The experimental results of the total target phenolic compound yield and antioxidant properties of the PHWE extracts of the *Bambusa chungii* culms followed the Box–Behnken design shown in Table 4. We generated four second-order equations to predict the total target phenolic compound yield, DPPH inhibition activity, ABTS inhibition activity, and ferric-reducing antioxidant power as follows:

Y_1_ = 3.48 + 0.44 A + 0.30 B + 0.49 C + 0.11 AB + 0.14 AC − 0.33 BC−0.07 A^2^ − 0.20 B^2^ − 1.00 C^2^ (5)

Y_2_ = 9.49 + 0.74 A + 0.29 B + 1.17 C + 0.26 AB + 0.29 AC − 0.30 BC−0.62 A^2^ − 0.91 B^2^ − 1.06 C^2^ (6)

Y_3_ = 38.96 + 5.41 A − 0.53 B + 10.86 C + 1.94 AB + 4.79 AC − 2.22 BC − 0.15 A^2^ − 0.57 B^2^ − 8.44 C^2^ (7)

Y_4_ = 1.00 − 0.06 A + 0.03 B + 0.12 C + 0.01 AB + 0.03 AC − 0.05 BC + 0.04 A^2^ − 0.04 B^2^ − 0.17 C^2^ (8)

In Equations (5)–(8), Y represents the dependent variables: Y_1_ was total yield of target phenolic compounds, Y_2_ was DPPH IC_50_**^−^**^1^, Y_3_ was ABTS IC_50_**^−^**^1^, and Y_4_ was ferric reducing antioxidant power. A, B and C correspond to the coded values of the three independent variables for material-to-liquid ratio, extraction time, and temperature, respectively.

ANOVA tests for each equation (Table S5) showed that all quadratic models were statistically significant, with *p*-values < 0.05. On the other hand, values from the lack-of-fit test for each model were >0.05, suggesting that the lack of fit, relative to pure error, was not significant. The quality of the fit of the second-order regression equation was evaluated using the coefficient of determination (*R*^2^), which was 0.9934, 0.8686, 0.9758, and 0.9606 for Y_1_, Y_2_ Y_3_, and Y_4_, respectively. The adjusted *R*^2^ values were 0.9848, 0.6996, 0.9448, and 0.9100, respectively, indicating that the developed models reliably predicted the dependent variables. The models showed good performance based on the observed and predicted values of the yield of the total target phenolic compounds, DPPH inhibition activity, ABTS inhibition activity, and the ferric-reducing antioxidant power.

The probability values of Y_1_, Y_2,_ Y_3_, and Y_4_ for single variables are presented in Table S5. The smaller the *p*-value, the more significant the corresponding coefficient. Based on the statistical results (ANOVA) with a confidence level of 95%, the temperature and material-to-liquid ratio had significant effects (*p* < 0.05) on the extraction of target phenolic compounds from *Bambusa chungii* culms, as well as the antioxidant capacity of the extracts.

**Figure 4:**
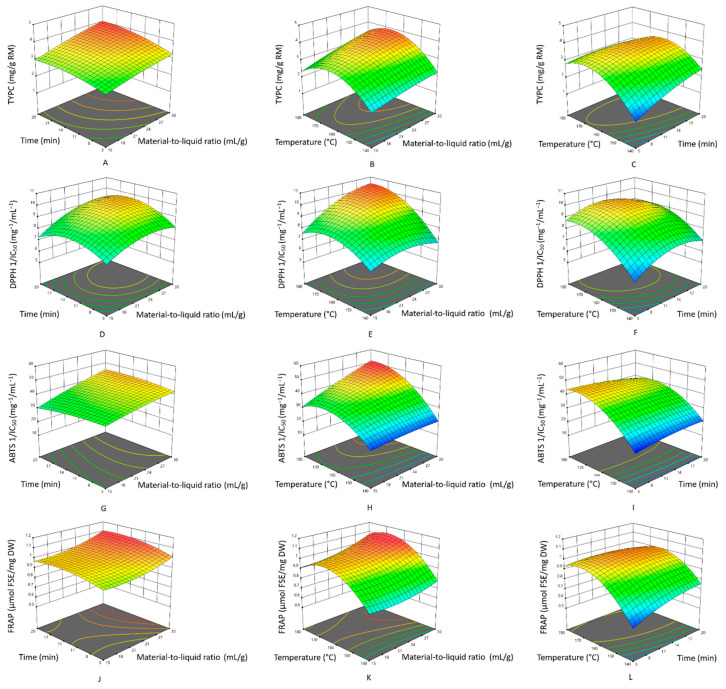
Response surface plots for the yield of the total target phenolic compounds (**A**–**C**) and antioxidant capacity measured by the DPPH (**D**–**F**), ABTS (**G**–**I**), and FRAP methods (**J**–**L**) as a function of the investigated factors.

Three-dimensional response surface curves were plotted using second-order equations to evaluate the combined effects of the variables. As shown in Figure 4A–C, a significant interaction between temperature and time on the yield of total target phenolic compounds was observed. The maximum yield of the total target phenolic compounds, 3.28 mg/g RM, was predicted at an extraction temperature of 165 °C and an extraction time of 14 min. Figure 4D–I show that higher extraction temperatures and lower material-to-liquid ratios achieved the highest DPPH and ABTS inhibition activities. Figure 4J–L shows the response surface plot for the ferric reducing antioxidant power. The pattern differed from that for the other two antioxidant assays, DPPH and ABTS. A high temperature and a long extraction time obtained the highest ferric-reducing antioxidant power. The target phenolic compounds possess different chemical structures that favor distinct extraction conditions, leading to diverse performances of the extracts in different antioxidant assays. These findings indicated that the PHWE extracts of *Bambusa chungii* culms with high DPPH and ABTS free radical scavenging activities might not be good ferric-reducing agents. The required antioxidant mechanism should be carefully considered during the optimization of extraction conditions for particular applications.

The suggested conditions for the optimal yield of the total target phenolic compounds and antioxidant activity of the PHWE extracts of the *Bambusa chungii* culms were 172.44 °C at 14.06 min and a material-to-liquid ratio of 1:30 g/mL. For operational convenience, the optimal parameters were slightly modified in the verification experiment as follows: 170 °C, 14 min, and a material-to-liquid ratio of 1:30 g/mL. We validated the optimal conditions by conducting triplicate PHWE experiments. For comparison, the results from the conventional solvent extractions are also tabulated in Table 5. The optimal PHWE extract exhibited the highest total yield of the target phenolic compounds (3.85 mg/g RM), approximately 39 and 7 times higher than that of extracts from maceration in ethanol and reflux extraction with ethanol, respectively. The antioxidant activity of the optimal PHWE extracts was also clearly stronger than that of the alcohol extracts.

There are very few results in the literature about the antioxidant capacity of *Bambusa chungii* culms. The data from the present work were compared to studies on the antioxidant capacity of the culms of other bamboo species. Wroblewska et al. employed the Soxhlet apparatus, with 60% (*v*/*v*) ethanol as the solvent, to extract natural antioxidants from five native Brazilian bamboo species [46]. Their IC_50_ values ranged from 168.18 to 244.36 μg/mL in the DPPH assay. The authors further investigated the in vitro photoprotective activity of the extracts, implying that the addition of bamboo culm extracts to sunscreen formulations significantly increased their Sun Protection Factor (SPF) and photostability. Choi et al. explored the application potential of bamboo culm extracts in cosmetics [68]. They evaluated the antioxidant and anti-melanogenic activities of Korean bamboo (*Phyllostachys nigra* var. henosis) culm extracts in vitro and in vivo. The best antioxidant results for the DPPH and ABTS tests were achieved by the ethyl acetate fraction of 80% (*v*/*v*) ethanol extract (DPPH IC_50_: 565.63 μg/mL and ABTS IC_50_: 414.61 μg/mL). These authors found that bamboo culm extracts demonstrated potent anti-melanogenic effects as an additive to whitening cosmetics. Tanaka et al. studied the biological activities and phytochemical profiles of extracts from different bamboo plant parts [69]. In their research, ten parts of *Phyllostachys pubescens* bamboo plants were subjected to extractions using ethanol and hot water. In the ABTS assay, the ethanol extract of the inner culm and the hot water extract of the outer culm showed the strongest antioxidant activities: the IC_50_ values were 88.5 and 113.7 μg/mL, respectively. The outer culm and inner culm were found to be abundant in phenolic compounds. Nuzul et al. evaluated the antioxidant capacity of extracts from *Bambusa beecheyana* Munro culms obtained by different extraction methods [33]. The IC_50_ of the maceration ethanol extract (72 h) and Soxhlet ethanol extract (1 h, repeated for six cycles) in the DPPH test were 95.93 and 87.12 μg/mL, respectively.

The above literature employed organic solvent extraction technologies, and the antioxidant activity evaluation methods were similar to those used in this work. By expressing all antioxidant activity values in the same units, we adequately compared our antioxidant capacity results with the literature values. In general, our optimal PHWE extract of the *Bambusa chungii* culms was superior to the literature data in terms of the antioxidant capacity. Regardless of the plant characteristics of different raw materials, this finding fully reflected the benefit of PHWE technology in recovering natural antioxidants from bamboo culms. More importantly, the PHWE used in this work had evident advantages from the beginning. In the era of sustainable development, PHWE is a feasible, environmentally friendly alternative to conventional extractions, as it is an organic solvent-free technique that consumes less time and energy.

## 4. Conclusions

In the present study, we successfully applied a pressurized hot water extraction to obtain natural phenolic antioxidants from *Bambusa chungii* culms. The operating conditions were optimized using a response surface method. Compared to conventional extractions, our study results demonstrated the efficiency of using pressurized hot water for a shorter extraction time, providing a higher extraction yield (22.61%) and higher total phenolic content (7.6 mg GAE/g RM). Furthermore, the LC–QTOF–MS analysis indicated a high-yield recovery of 19 target phenolic compounds in the PHWE extracts. The evaluations of single parameter effects validated that temperature was the most influential factor in the total yield of the target phenolic compounds. Furthermore, an appropriate elevated temperature resulted in a markedly increased TYPC rather than the highest temperature explored in this research. The extraction temperature also significantly affected the antioxidant capacity of the PHWE extracts. Statistical analysis confirmed that the antioxidant activity of the *Bambusa chungii* culms measured by DDPH, ABTS, and FRAP assays strongly correlated with the total yield of the target phenolic compounds. Furthermore, the extraction time and material-to-liquid ratio also affected the TYPC and antioxidant activity of the PHWE extracts. A comparatively short extraction time and a moderate material-to-liquid ratio are recommended for the PHWE process. The established second-order model was suitable for predicting the studied responses of the TYPC and antioxidant capacity of the extracts. The optimum extraction parameters were found as 170 °C, with a 1:30 g/mL material-to-liquid ratio and a 14 min extraction time. The PHWE extract of the *Bambusa chungii* culms obtained at optimal conditions showed a markedly high TYPC level (3.85 mg/g RM) and antioxidant capacity (IC_50_ of DPPH test: 94.7 mg/L, IC_50_ of ABTS test: 21.88 mg/L, and the FRAP value: 1.23 μmol FSE/mg of dried extract) compared with conventional organic solvent extractions; and presented application potential in different fields, such as foods, pharmaceuticals, or cosmetics. Therefore, our results demonstrated the importance of using a promising pressurized hot water extraction method to recover valuable natural antioxidants from a widely distributed and fast-growing raw material, *Bambusa chungii* culms.

## Figures and Tables

**Table 1:** Comparisons between the extraction yield, TPC, OD_420_ value, and contents of FF and HMF in the extracts obtained from PHWE at different temperatures and from conventional extractions.

Extracts	Extraction Yield (%)	TPC (mg GAE/g RM)	Browning Intensity (OD_420_)	FF µg/g RM	HMF µg/g RM
Maceration	3.31 ± 0.06 ^a^	1.29 ± 0.22 ^a^	0.28 ± 0.00 ^a^	ND	ND
Reflux	7.1 ± 0.4 ^b^	2.56 ± 0.15 ^c^	0.50 ± 0.02 ^b^	ND	ND
PHWE 100 °C	7.60 ± 0.21 ^c^	1.58 ± 0.05 ^b^	0.30 ± 0.02 ^a^	ND	ND
PHWE 120 °C	8.75 ± 0.17 ^d^	2.0 ± 0.5 ^bc^	0.30 ± 0.01 ^a^	ND	ND
PHWE 140 °C	9.2 ± 0.7 ^d^	3.28 ± 0.06 ^d^	0.39 ± 0.02 ^a^	ND	69 ± 6 ^a^
PHWE 160 °C	14.18 ± 0.09 ^e^	5.11 ± 0.10 ^e^	0.54 ± 0.01 ^b^	221 ± 16 ^a^	523 ± 25 ^b^
PHWE 180 °C	22.6 ± 0.7 ^f^	7.6 ± 0.4 ^f^	0.72 ± 0.11 ^c^	2805 ± 97 ^b^	2246 ± 36 ^c^

Results are expressed as mean ± standard deviations (SD) (*n* = 3). Values represented with different lowercase letters are statistically significant differences (*p* < 0.05). TPC: total phenolic content, OD_420_: optical density measured at 420 nm, FF: furfural, HMF: 5-hydroxymethylfurfural, PHWE: pressurized hot water extraction, ND: Not detected, RM: raw material of *Bambusa chungii* culms.

**Table 2:** Contents of target phenolic compounds in the extracts obtained from PHWE at different temperatures and from conventional extractions.

Compounds	Maceration (µg/g RM)	Reflux (µg/g RM)	PHWE (µg/g RM)				
			100 °C	120 °C	140 °C	160 °C	180 °C
Quinic acid	11.32 ± 0.25 ^b^	18.4 ± 1.1 ^c^	17.48 ± 0.29 ^c^	19.4 ± 0.7 ^cd^	20.8 ± 1.3 ^d^	20.6 ± 0.6 ^d^	23.8 ± 1.7 ^e^
Arbutin	ND	ND	7.5 ± 1.2 ^a^	14.4 ± 0.8 ^c^	ND	ND	ND
Adenosine	3.6 ± 0.7 ^a^	9.0 ± 0.5 ^b^	46 ± 6 ^f^	31.9 ± 1.6 ^e^	25.8 ± 1.0 ^d^	25.61 ± 0.15 ^d^	12.2 ± 0.3 ^c^
Tachioside	ND	ND	ND	10.8 ± 0.3 ^a^	ND	ND	ND
Vanillic alcohol	ND	ND	4.39 ± 0.20 ^a^	11.0 ± 1.3 ^b^	23.1 ± 1.5 ^c^	ND	ND
*p*-Hydroxybenzoic acid	6.99 ± 0.13 ^a^	12.0 ± 0.4 ^b^	5.9 ± 0.9 ^a^	13.7 ± 0.8 ^c^	16.0 ± 0.7 ^d^	27.8 ± 0.6 ^f^	35.6 ± 1.9 ^g^
Vanillic acid	39.6 ± 1.0 ^c^	66.4 ± 0.5 ^e^	29.5 ± 2.1 ^a^	29.0 ± 1.1 ^a^	36.7 ± 1.8 ^b^	54.3 ± 2.0 ^d^	85.7 ± 1.2 ^f^
*p*-Hydroxybenzaldehyde	9.5 ± 0.4 ^ab^	29.6 ± 1.0 ^ab^	ND	35 ± 4 ^b^	192 ± 4 ^c^	318 ± 38 ^d^	384 ± 12 ^e^
Caffeic acid	ND	ND	ND	13.5 ± 1.3 ^c^	9.7 ± 0.4 ^b^	7.0 ± 0.6 ^a^	6.5 ± 0.6 ^a^
Syringic acid	9.5 ± 0.4 ^ab^	10.3 ± 0.4 ^ab^	11.76 ± 0.23 ^ab^	13.6 ± 1.8 ^b^	21.6 ± 2.4 ^c^	56.6 ± 2.8 ^d^	111 ± 9 ^e^
Vanillin	ND	5.3 ± 0.4 ^a^	ND	6.7 ± 0.6 ^a^	27.6 ± 2.7 ^b^	113 ± 6 ^c^	181 ± 6 ^d^
*p*-Coumaric acid	7.7 ± 0.4 ^a^	237.0 ± 2.0 ^b^	51 ± 5 ^a^	254 ± 21 ^b^	896 ± 58 ^c^	1145 ± 39 ^e^	1081 ± 49 ^d^
Coniferol	ND	39.4 ± 0.6 ^b^	10.7 ± 1.5 ^ab^	76 ± 7 ^c^	736 ± 21 ^d^	942 ± 38 ^e^	49.5 ± 2.7 ^bc^
Syringaldehyde	ND	7.4 ± 0.5 ^b^	5.10 ± 0.23 ^ab^	7.42 ± 0.27 ^b^	22.2 ± 2.8 ^c^	81.3 ± 2.0 ^d^	170 ± 5 ^e^
Ferulic acid	ND	4.6 ± 0.4 ^a^	3.29 ± 0.18 ^a^	5.4 ± 0.4 ^a^	22 ± 4 ^b^	39 ± 4 ^c^	85 ± 4 ^d^
Coniferylaldehyde	ND	ND	ND	ND	25.7 ± 2.0 ^a^	60.80 ± 0.27 ^b^	111 ± 11 ^c^
Sinapaldehyde	ND	8.0 ± 0.7 ^b^	ND	3.2 ± 0.6 ^a^	26 ± 3 ^c^	57.3 ± 0.8 ^d^	98 ± 5 ^e^
Syringaresinol	8.5 ± 1.1 ^a^	35.8 ± 1.5 ^b^	10.8 ± 0.7 ^a^	63.8 ± 0.6 ^c^	276 ± 7 ^e^	519.4 ± 1.2 ^f^	210 ± 16 ^d^
Balanophonin	ND	3.3 ± 0.3 ^a^	ND	5.3 ± 0.4 ^b^	5.50 ± 0.19 ^b^	7.7 ± 1.1 ^c^	12.4 ± 1.9 ^d^
Total yield	96.9 ± 2.3 ^a^	486 ± 4 ^b^	202.8 ± 1.7 ^a^	614 ± 25 ^c^	2391 ± 72 ^d^	3470 ± 112 ^f^	2644 ± 22 ^e^

Results are expressed as mean ± SD (*n* = 3). Values represented with different lowercase letters are statistically significant differences (*p* < 0.05). ND: Not detected.

**Table 3:** Pearson correlation test for the TYPC and antioxidant activity of PHWE extracts obtained at different temperatures.

Parameter	DPPH	ABTS	FRAP	TYPC
DPPH	1			
ABTS	0.934 *	1		
FRAP	−0.866	−0.816	1	
TYPC	−0.978 **	−0.928 *	0.949 *	1

** indicated extremely significant correlation (*p* < 0.01); * represents significant correlation (*p* < 0.05).

**Table 4:** Independent variables and experimental data of the response design for the PHWE of phenolic antioxidants from *Bambusa chungii* culms.

Run	Independent Variables			Dependent Variables: Experimental Values			
	A (mL/g)	B (min)	C (°C)	Y_1_ (mg/g RM)	Y_2_ (mg^−1^/mL^−1^)	Y_3_ (mg^−1^/mL^−1^)	Y_4_ (μmol FSE/mg DW)
1	20	20	140	2.29	7.34	19.32	0.77
2	20	5	180	2.64	7.40	41.65	0.88
3	30	10	180	3.35	10.71	50.71	1.09
4	20	10	160	3.26	8.95	46.47	1.00
5	20	10	160	3.09	9.23	38.93	1.00
6	20	10	160	3.24	9.68	38.77	1.01
7	30	20	160	4.08	8.92	45.68	1.08
8	30	5	160	3.34	8.05	42.88	1.01
9	15	20	160	2.90	7.01	30.33	0.95
10	15	10	140	1.51	5.60	20.12	0.66
11	30	10	140	1.89	6.26	18.50	0.75
12	20	20	180	2.53	8.06	33.98	0.89
13	15	10	180	2.40	7.87	32.59	0.91
14	20	5	140	1.06	6.02	17.56	0.57
15	15	5	160	2.53	7.85	34.04	0.97
16	20	10	160	3.22	8.90	35.73	0.92
17	20	10	160	3.28	8.23	33.43	0.94

A, material-to-liquid ratio; B, extraction time; C, temperature; Y_1_, total yield of target phenolic compounds; Y_2_, DPPH IC_50_**^−^**^1^; Y_3_, ABTS IC_50_**^−^**^1^; Y_4_, ferric reducing antioxidant power.

**Table 5:** Comparison of the TYPC and each antioxidant property between predicted values from mathematical models, actual experiment results of the optimal PHWE and conventional solvent extracts.

Extracts		TYPC (mg/g RM)	DPPH IC_50_ (mg/L)	ABTS IC_50_ (mg/L)	FRAP (μmol FSE/mg DW)
Predicted values	172.44 °C, 1:30 g/mL, 14.06 min	3.81 ± 0.08	96.3 ± 1.4	19.74 ± 0.26	1.12 ± 0.04
Experimental values	170 °C, 1:30 g/mL, 14 min	3.85 ± 0.01	94.7 ± 0.6	21.88 ± 0.16	1.23 ± 0.02
Maceration	80% ethanol, 1:20 g/mL, 72 h × 2	0.096 ± 0.002	322 ± 5	43.0 ± 0.4	0.41 ± 0.02
Reflux extraction	80% ethanol, 1:30 g/mL 2 h × 3	0.485 ± 0.004	189 ± 5	26.84 ± 0.06	0.88 ± 0.01

Results are expressed as mean ± SD (*n* = 3).

## Data Availability

Data is contained within the article and the Supplementary Materials.

## Supplementary Materials

The following supporting information can be downloaded at: https://www.mdpi.com/article/10.3390/antiox11112231/s1, Table S1: Elution gradient. Table S2: MS data and the identification results of the target phenolic compounds in the *Bambusa chungii* culms extracts by LC-QTOF-MS. Table S3: Contents of the target phenolic compounds, FF, and HMF in the extracts obtained from PHWE at different extraction time. Table S4: Contents of the target phenolic compounds, FF, and HMF in the extracts obtained from PHWE at different material-to-liquid ratio. Table S5: Model summary and ANOVA results of the response variables. Figure S1: Schematic of PHWE extraction. Figure S2: Color of the *Bambusa chungii* culms extracts obtained by PHWE and conventional extraction methods. Figure S3: Chemical structures of the extracted target phenolic compounds.
