# Supplementary material for: High-Yield Recovery of Antioxidant Compounds from Bambusa chungii Culms Using Pressurized Hot Water Extraction
Source: Antioxidants (Basel). 2022 Nov 12;11(11):2231. doi: 10.3390/antiox11112231 (PMC9686541; doi:10.3390/antiox11112231)

Figure S2. Color of the *Bambusa chungi*i culms extracts obtained by PHWE and conventional extraction methods.

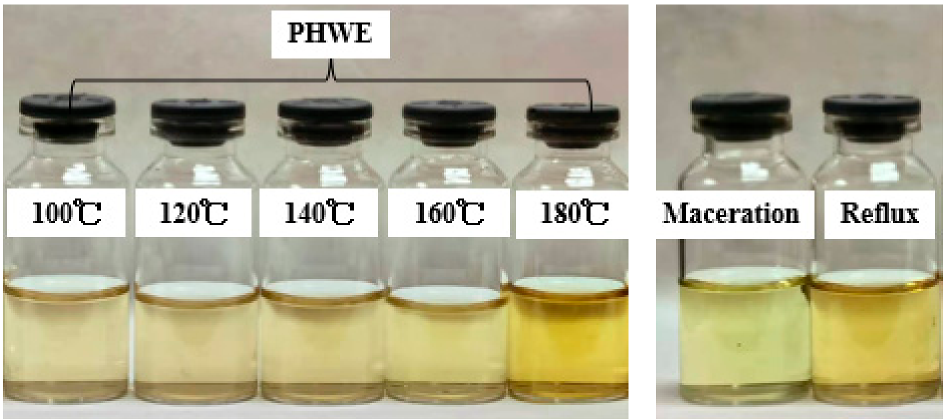

Supplement: Supplementary file 1 [file antioxidants-11-02231-s001.zip › Figure S2. Color of the Bambusa chungii culms extracts obtained by PHWE and conventional extraction methods..pdf]
