# Supplementary material for: High-Yield Recovery of Antioxidant Compounds from Bambusa chungii Culms Using Pressurized Hot Water Extraction
Source: Antioxidants (Basel). 2022 Nov 12;11(11):2231. doi: 10.3390/antiox11112231 (PMC9686541; doi:10.3390/antiox11112231)
Supplement: Supplementary file 1 [file antioxidants-11-02231-s001.zip › Table S1. Elution gradient.pdf]

Table S1. Elution gradient

Time (min)	Mobile phase composition	
	A (%)	B (%)
0	95	5
5	95	5
7	92	8
20	90	10
35	75	25
45	60	40
50	10	90
55	10	90
