# Supplementary material for: High-Yield Recovery of Antioxidant Compounds from Bambusa chungii Culms Using Pressurized Hot Water Extraction
Source: Antioxidants (Basel). 2022 Nov 12;11(11):2231. doi: 10.3390/antiox11112231 (PMC9686541; doi:10.3390/antiox11112231)
Supplement: Supplementary file 1 [file antioxidants-11-02231-s001.zip › Table S2. MS data and identification results of the target phenolic compounds in Bambusa chungii culms extracts by LC-Q-TOF-MS..pdf]

Table S2. MS data and identification results of the target phenolic compounds in *Bambusa chungii* culms extracts by LC-Q-TOF-MS.

No.	Retention time (min)	Species	Measured （m/z）	Calculated （m/z）	Error （ppm）	Main fragments	Formula	Identification
1	1.53	[M+H]+	193.0702	193.0707	0.25	147.0650	$C_7H_{12}O_6$	Quinic acid
2	2.07	[M+Na]+	295.0783	295.0788	2.11	176.5382	$C_{12}H_{16}O_7$	Arbutin
3	2.08	[M+H]+	268.1026	268.104	-2.08	136.0636	$C_{10}H_{13}N_5O_4$	Adenosine
4	3.05	[M+Na]+	325.0897	325.0894	-0.65	171.0289	$C_{13}H_{18}O_8$	Tachioside
5	4.4	[M+H]+	127.0388	127.0390	1.45	109.0279	$C_6H_6O_3$	5-Hydroxymethylfurfural*
6	6.12	[M+H]+	97.0284	97.0284	-3.18	-	$C_5H_4O_2$	Furfural *
7	8.44	[M+H-H2O]+	137.0593	137.0597	2.96	-	$C_8H_{10}O_3$	Vanillic alcohol
8	9.73	[M+H]+	139.0386	139.0390	-2.26	121.0292	$C_7H_6O_3$	*p*-Hydroxybenzoic acid
9	12.81	[M+H]+	169.0492	169.0495	3.44	-	$C_8H_8O_4$	Vanillic acid
10	13.31	[M+H]+	123.0437	123.0441	2.89	95.0482	$C_7H_6O_2$	*p*-Hydroxybenzaldehyde
11	13.37	[M+H]+	181.0491	181.0495	2.54	163.037	$C_9H_8O_4$	Caffeic acid
12	15.3	[M+H]+	199.0595	199.0601	1.21	155.0681	$C_9H_{10}O_5$	Syringic acid
13	18.34	[M+H]+	153.0541	153.0546	0.75	125.063	$C_8H_8O_3$	Vanillin
14	20.68	[M+H]+	165.0540	165.0546	1.14	147.0441	$C_9H_8O_3$	*p*-Coumaric acid
15	21.33	[M+H-H2O]+	181.0858	181.0859	-2.11	131.0493	$C_{10}H_{12}O_3$	Coniferol
16	23.1	[M+H]+	183.0649	183.0652	0.84	155.0714	$C_9H_{10}O_4$	Syringaldehyde
17	25.96	[M+H]+	195.0648	195.0652	-0.63	177.0555	$C_{10}H_{10}O_4$	Ferulic acid
18	29.89	[M+H]+	179.0699	179.0703	-1.08	151.0377	$C_{10}H_{10}O_3$	Coniferylaldehyde
19	31.13	[M+H]+	209.0804	209.0808	-1.25	165.0469	$C_{11}H_{12}O_4$	Sinapaldehyde
20	39.52	[M+H]+	419.1697	419.1700	1.27	265.1074	$C_{22}H_{26}O_8$	Syringaresinol
21	40.42	[M+H]+	357.1328	357.1333	0.48	339.1206	$C_{20}H_{20}O_6$	Balanophonin

All the identified compounds were compared with standard compounds.

*: 5-Hydroxymethylfurfural (HMF) and furfural (FF) were deteted by this method.
