# Supplementary material for: High-Yield Recovery of Antioxidant Compounds from Bambusa chungii Culms Using Pressurized Hot Water Extraction
Source: Antioxidants (Basel). 2022 Nov 12;11(11):2231. doi: 10.3390/antiox11112231 (PMC9686541; doi:10.3390/antiox11112231)
Supplement: Supplementary file 1 [file antioxidants-11-02231-s001.zip › Table S3. Contents of the target phenolic compounds, FF, and HMF in the extracts obtained from PHWE at different extraction time..pdf]

Table S3. Contents of the target phenolic compounds, FF, and HMF in the extracts obtained from PHWE at different extraction time.

Compounds	Yield (µg/g RM)				
	5 min	10 min	20 min	30 min	40 min
Quinic acid	20.5±0.6[bc]	23.0±1.0[c]	20.8±0.9[b]	18.9±0.4[b]	15.2±1.6[a]
Adenosine	34.5±0.3[c]	27.80±0.22[b]	25.61±0.15[b]	22.5±1.3[a]	26.4±2.1[b]
Arbutin	ND	ND	ND	ND	ND
Tachioside	ND	ND	ND	ND	ND
Vanillic alcohol	ND	ND	ND	ND	ND
p-Hydroxybenzoic acid	23.53±0.27[a]	23.64±0.29[a]	27.8±1.4[b]	27.3±0.4[b]	25.7±1.7[ab]
Vanillic acid	109±6[b]	75.4±0.5[b]	59.2±0.3[a]	58.7±2.4[a]	51±10[a]
p-Hydroxybenzaldehyde	245.5±1.5[a]	254±5[a]	304±5[b]	331±3[c]	329±4[c]
Caffeic acid	10.90±0.04[a]	10.6±0.8[a]	10.91±0.10[a]	10.3±0.4[a]	11.5±1.3[a]
Syringic acid	39±7[a]	39.3±2.3[a]	53.8±1.8[b]	54±4[b]	53±6[b]
Vanillin	59.60±0.15[a]	70±7[b]	105±3[b]	110.0±1.3[c]	112.8±0.5[c]
p-Coumaric acid	997±49[a]	1098±20[b]	1122±31[c]	1230±13[c]	1059±20[d]
Coniferol	1112±39[d]	1060±27[d]	976±8[c]	838±31[b]	730±19[a]
Syringaldehyde	49.0±0.3[a]	47.1±1.0[a]	90.5±0.5[b]	97±5[bc]	103±8[c]
Ferulic acid	30.1±0.7[a]	32.7±0.5[a]	44.2±0.5[b]	49.30±0.09[c]	46.7±2.7[bc]
Coniferylaldehyde	51±3[a]	49.11±0.10[a]	72.6±0.4[b]	68±6[b]	67.4±0.8[b]
Sinapaldehyde	44±4[a]	44.7±2.9[a]	53.2±1.1[b]	70.45±0.26[c]	68±4[c]
Syringaresinol	455.3±0.8[a]	462±10[a]	519.4±1.2[c]	523.1±2.0[c]	499±10[b]
Balanophonin	8.15±0.01[a]	8.7±0.7[a]	10.19±0.03[b]	11.31±0.13[c]	8.2±0.3[a]
TYPC	3287±13[b]	3326±37[b]	3496±20[c]	3520±56[c]	3204.490±0.002[a]
HMF	227.0±1.9[a]	313±5[b]	555±6[c]	791±16[d]	726±15[e]
Furfural	288.1±1.6[a]	292±3[a]	313±8[a]	412±5[b]	472±7[b]

Results are expressed as mean ± SD (n=3). Values represented with different lowercase letters are statistically significant differences ($p < 0.05$). RM: raw material. ND: Not detected.
