# Supplementary material for: High-Yield Recovery of Antioxidant Compounds from Bambusa chungii Culms Using Pressurized Hot Water Extraction
Source: Antioxidants (Basel). 2022 Nov 12;11(11):2231. doi: 10.3390/antiox11112231 (PMC9686541; doi:10.3390/antiox11112231)
Supplement: Supplementary file 1 [file antioxidants-11-02231-s001.zip › Table S4. Contents of the target phenolic compounds, FF, and HMF in the extracts obtained from PHWE at different material-to-liquid ratio..pdf]

Table S4. Contents of the target phenolic compounds, FF, and HMF in the extracts obtained from PHWE at different material-to-liquid ratio.

Compounds	Yield (µg/g RM)				
	**1:30 (g/mL)**	**1:20 (g/mL)**	**1:15 (g/mL)**	**1:10 (g/mL)**	**1:5 (g/mL)**
Quinic acid	24.9±1.0^{b}	20.8±0.9^{a}	22.6±0.4^{b}	19.2±0.8^{a}	21.4±1.6^{a}
Adenosine	27.9±2.2^{bc}	25.61±0.15^{b}	29.77±0.10^{c}	24.45±0.25^{b}	16.6±1.4^{a}
Arbutin	ND	ND	ND	ND	ND
Tachioside	ND	ND	ND	ND	ND
Vanillic alcohol	ND	ND	ND	4.21±0.27^{a}	3.33±0.15^{b}
p-Hydroxybenzoic acid	33.97±0.28^{c}	27.8±1.4^{b}	24.6±1.3^{b}	22±4^{ab}	18.5±1.0^{a}
Vanillic acid	63.4±1.0^{e}	59.2±0.3^{d}	46.0±0.8^{c}	35.31±0.28^{b}	32.6±0.8^{a}
p-Hydroxybenzaldehyde	346±4^{d}	304±5^{c}	263±4^{b}	236±20^{a}	215±10^{a}
Caffeic acid	9.7±0.4^{c}	10.91±0.10^{d}	8.57±0.27^{b}	8.38±0.23^{b}	3.09±0.17^{a}
Syringic acid	58.90±0.09^{c}	53.8±1.8^{b}	55.0±0.4^{b}	37.3±1.0^{a}	39.3±1.9^{a}
Vanillin	120.5±1.9^{e}	105±3^{d}	80.5±2.1^{c}	53.94±0.12^{b}	38.3±0.8^{a}
p-Coumaric acid	1139±11^{d}	1122±31^{d}	942±19^{c}	864±12^{b}	758±32^{a}
Coniferol	1430±33^{d}	966±7^{c}	978±22^{c}	716±11^{b}	584±12^{a}
Syringaldehyde	98.8±2.5^{e}	90.5±0.5^{d}	72.4±0.3^{c}	53.2±1.0^{b}	32.2±1.6^{a}
Ferulic acid	40.7±0.3^{c}	44.2±0.5^{c}	35.6±1.2^{b}	24.89±0.05^{a}	23.7±2.7^{a}
Sinapic acid	ND	ND	ND	3.83±0.07^{b}	2.8±0.4^{a}
Coniferylaldehyde	78.2±0.8^{d}	72.6±0.4^{d}	55±5^{c}	38.9±0.9^{b}	29.0±1.5^{a}
Sinapaldehyde	61.36±0.13^{d}	53.2±1.1^{c}	41±5^{b}	29.3±2.2^{a}	24.9±1.9^{a}
Syringaresinol	581±13^{e}	519.4±1.2^{d}	346±7^{c}	227.6±0.4^{b}	201±6^{a}
Balanophonin	11.6±0.6^{e}	10.19±0.03^{d}	7.8±0.6^{c}	5.7±0.3^{b}	3.3±0.3^{a}
TYPC	4119±40^{e}	3486±34^{d}	3009±54^{c}	2409±31^{b}	2050±56^{a}
HMF	532±3^{b}	555±6^{b}	536±13^{b}	379±16^{a}	399±32^{a}
Furfural	168±14^{a}	313±8^{c}	341±4^{d}	232±8^{b}	245±15^{b}

Results are expressed as mean ± SD (n=3). Values represented with different lowercase letters are statistically significant differences ($p < 0.05$). ND: Not detected.
