# Supplementary material for: High-Yield Recovery of Antioxidant Compounds from Bambusa chungii Culms Using Pressurized Hot Water Extraction
Source: Antioxidants (Basel). 2022 Nov 12;11(11):2231. doi: 10.3390/antiox11112231 (PMC9686541; doi:10.3390/antiox11112231)
Supplement: Supplementary file 1 [file antioxidants-11-02231-s001.zip › Table S5. Model summary and ANOVA results of the response variables.pdf]

Table S5. Model summary and ANOVA results of the response variables

Source	Sum of Squares				Mean Square				F-value				P-value			
	Y_1	Y_2	Y_3	Y_4	Y_1	Y_2	Y_3	Y_4	Y_1	Y_2	Y_3	Y_4	Y_1	Y_2	Y_3	Y_4
Model	9.13	25.03	1540.50	0.31	1.01	2.78	171.17	0.04	116.49	5.14	31.41	18.97	< 0.0001	0.0211	< 0.0001	0.0004
A	1.43	4.22	223.00	0.02	1.43	4.22	223.00	0.02	163.97	7.81	40.92	12.84	< 0.0001	0.0268	0.0004	0.0089
B	0.6707	0.6608	2.12	9.20 x10^{-3}	0.6707	0.6608	2.12	9.20 x10^{-3}	77.00	1.22	0.3897	4.96	< 0.0001	0.3056	0.5523	0.0611
C	1.72	9.79	848.79	0.11	1.72	9.79	848.79	0.11	197.81	18.09	155.76	59.17	< 0.0001	0.0038	< 0.0001	0.0001
AB	0.0515	0.2993	16.74	6.00 x10^{-4}	0.0515	0.2993	16.74	6.00 x10^{-4}	5.91	0.5532	3.07	0.32	0.0453	0.4812	0.1231	0.5867
AC	0.0808	1.96	96.68	2.80 x10^{-3}	0.0808	1.96	96.68	2.80 x10^{-3}	9.27	3.63	17.74	1.52	0.0187	0.0985	0.0040	0.2576
BC	0.4613	0.3859	20.69	0.01	0.4613	0.3859	20.69	0.01	52.97	0.7133	3.80	6.18	0.0002	0.4263	0.0924	0.0418
A^2	0.0155	1.21	0.0726	4.10 x10^{-3}	0.0155	1.21	0.0726	4.10 x10^{-3}	1.77	2.24	0.0133	2.21	0.2245	0.1785	0.9114	0.1804
B^2	0.1210	2.59	1.03	4.20 x10^{-3}	0.1210	2.59	1.03	4.20 x10^{-3}	13.89	4.78	0.1895	2.30	0.0074	0.0650	0.6764	0.1735
C^2	4.20	4.68	298.84	0.13	4.20	4.68	298.84	0.13	482.53	8.65	54.84	68.28	< 0.0001	0.0217	0.0001	< 0.0001
Residual	0.0610	3.79	38.15	0.01	0.0087	0.5410	5.45	1.80 x10^{-3}								
Lack of fit	0.0385	2.67	6.00	6.90 x10^{-3}	0.0128	0.8889	2.00	2.30 x10^{-3}	2.28	3.17	0.2490	1.52	0.2210	0.1470	0.8587	0.3383
Pure error	0.0225	1.12	32.14	6.00 x10^{-3}	0.0056	0.2802	8.04	1.50 x10^{-3}								
Total	9.19	28.82	1578.64	0.33												

Y_1: R^2=0.9941, R^2_{Adj}=0.9864, Ratio=41.2781, C.V.%=3.16%; Y_2: R^2=0.8686, R^2_{Adj}=0.6996, Ratio=7.9085, C.V.%=9.19%

Y_3: R^2=0.9758, R^2_{Adj}=0.9448, Ratio =18.8828, C.V.%=6.91%; Y_4: R^2=0.9606, R^2_{Adj}=0.9100, Ratio =15.8843, C.V.%=4.74%

ANOVA: Analysis of variance; R^2: coefficient of determination; R^2_{Adj}: the adjusted R^2; Ratio: signal to noise ratio; C.V.: coefficients of variation.
