# Supplementary figures and images for: High-Yield Recovery of Antioxidant Compounds from Bambusa chungii Culms Using Pressurized Hot Water Extraction
Source: Antioxidants (Basel). 2022 Nov 12;11(11):2231. doi: 10.3390/antiox11112231 (PMC9686541; doi:10.3390/antiox11112231)

**Figure S1.** Schematic of PHWE extraction

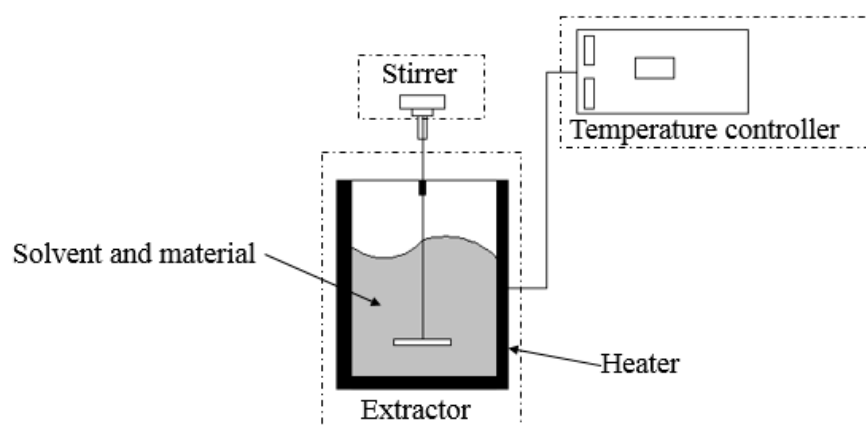

Supplement: Supplementary file 1 [file antioxidants-11-02231-s001.zip › Figure S1. Schematic of PHWE extraction.pdf]

**Figure S3.** Chemical structures of the extracted target phenolic compounds

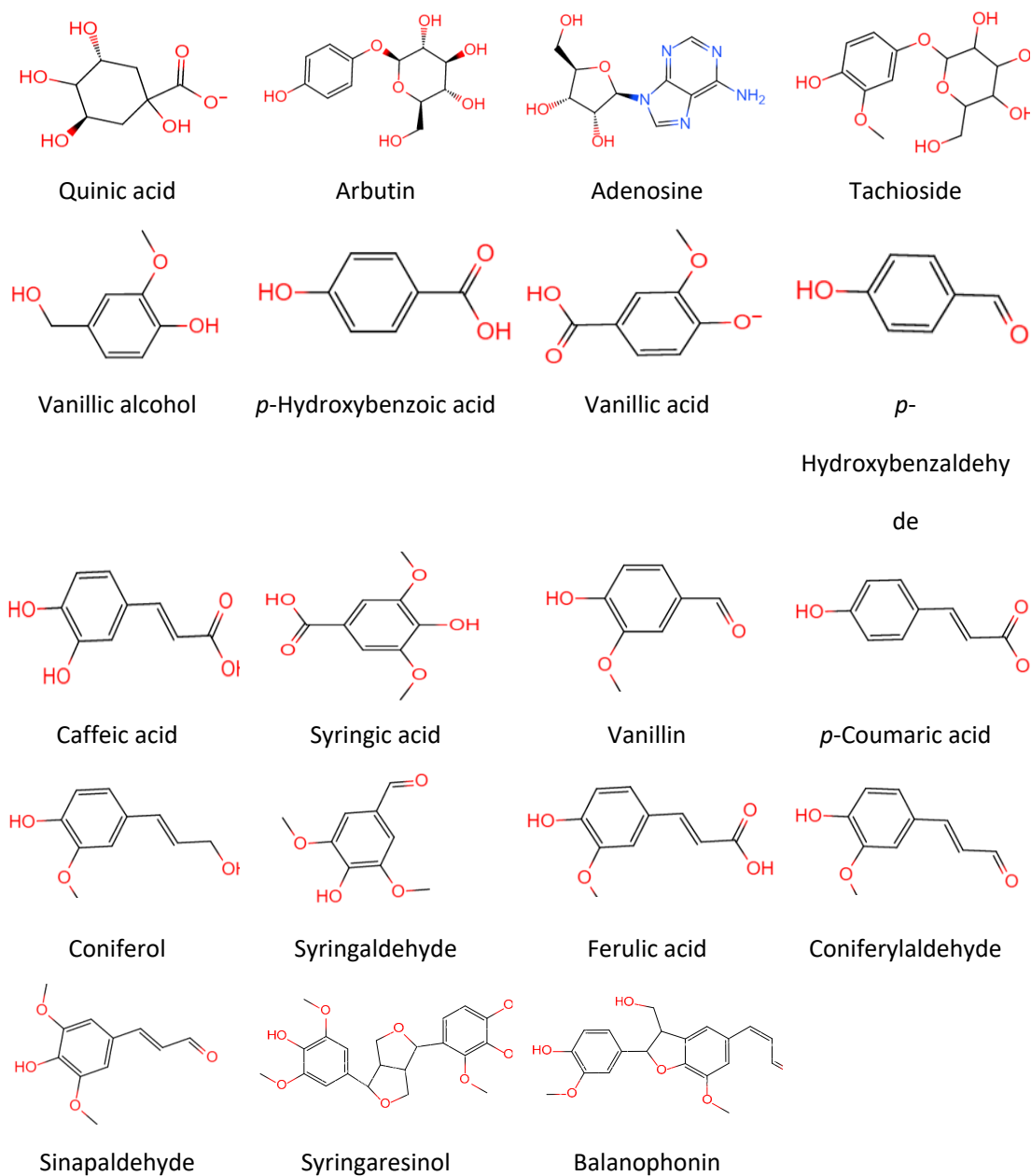

Supplement: Supplementary file 1 [file antioxidants-11-02231-s001.zip › Figure S3. Chemical structures of the extracted target phenolic compounds.pdf]
